# The Neuromodulatory Impact of Subthalamic Nucleus Deep Brain Stimulation on Gait and Postural Instability in Parkinson's Disease Patients: A Prospective Case Controlled Study

**DOI:** 10.3389/fneur.2018.00906

**Published:** 2018-10-31

**Authors:** Stanislaw Szlufik, Maria Kloda, Andrzej Friedman, Iwona Potrzebowska, Kacper Gregier, Tomasz Mandat, Andrzej Przybyszewski, Justyna Dutkiewicz, Monika Figura, Piotr Habela, Dariusz Koziorowski

**Affiliations:** ^1^Department of Neurology, Faculty of Health Science, Medical University of Warsaw, Warsaw, Poland; ^2^Department of Rehabilitation, II Faculty of Medicine, Medical University of Warsaw, Warsaw, Poland; ^3^Department of Neurosurgery, Maria Sklodowska Curie Memorial Oncology Center, Warsaw, Warsaw, Poland; ^4^Department of Informatics, Polish Japanese Academy of Information Technology, Warsaw, Poland

**Keywords:** DBS (deep brain stimulation), neuromodulation, Parkinson's disease, gait, instability analysis

## Abstract

**Background:** Subthalamic nucleus deep brain stimulation (STN-DBS) has been an established method in improvement of motor disabilities in Parkinson's disease (PD) patients. It has been also claimed to have an impact on balance and gait disorders in PD patients, but the previous results are conflicting.

**Objective:** The aim of this prospective controlled study was to evaluate the impact of STN-DBS on balance disorders in PD patients in comparison with Best-Medical-Therapy (BMT) and Long-term-Post-Operative (POP) group.

**Methods:** DBS-group consisted of 20 PD patients (8F, 12M) who underwent bilateral STN DBS. POP-group consisted of 14 post-DBS patients (6F, 8M) in median 30 months-time after surgery. Control group (BMT-group) consisted of 20 patients (11F, 9M) who did not undergo surgical intervention. UPDRS III scale and balance tests (Up And Go Test, Dual Task- Timed Up And Go Test, Tandem Walk Test) and posturography parameters were measured during 3 visits in 9 ± 2months periods (V1, V2, V3) 4 phases of treatment (BMT-ON/OFF, DBS-ON/OFF).

**Results:** We have observed the slowdown of gait and postural instability progression in first 9 post-operative months followed by co-existent enhancement of balance disorders in next 9-months evaluation (*p* < 0.05) in balance tests (Up and Go, TWT) and in posturography examination parameters (*p* < 0.05). The effect was not observed neither in BMT-group nor POP-group (*p* > 0.05): these groups revealed constant progression of static and dynamic instability (*p* > 0.05).

**Conclusions:** STN-DBS can have modulatory effect on static and dynamic instability in PD patients: it can temporarily improve balance disorders. mainly during first 9 post-operative months, but with possible following deterioration of the symptoms in next post-operative months.

## Introduction

Parkinson's disease (PD) in one of the most common neurodegenerative disorders with dominating motor symptoms such as bradykinesia, tremor and rigidity (1). The progression of the disease is often related to balance disorders and therefore can be a reason of falls with secondary injuries and increased possibility of hip fractures (2). Therefore the complex assessment of gait and postural instability in PD patients is crucial and can have a serious impact on quality of life in this group of patients (3).

Subthalamic nucleus deep brain stimulation (STN-DBS) has been a standard surgical procedure for PD patients with adverse effects after levodopa treatment or with motor fluctuations irrespective of best medical treatment (BMT) (4). STN-DBS has been shown to influence in addition to tremor, rigidity, and bradykinesia, also improves gait speed, step length and reduces gait variability (better postural control) (5–11). However, long-term observation of STN-DBS effect on balance disorders is not so clear, as some authors described the improvement in postural instability and gait difficulties only in first post-operative months after STN-DBS, but not in long-term assessment (12–14). To make these evidences more conflicting, there are also studies suggesting the aggravation of postural instability in PD patients after DBS (15).

Therefore, the aim of the study was to evaluate the impact of STN-DBS on gait and postural instability in PD patients in comparison with Best Medical Therapy (BMT) and Long-term Post-Operative (POP) groups.

## Materials and methods

The study cohort consisted of clinically diagnosed as idiopathic Parkinson's disease patients that fulfilled UK Parkinson's Disease Society (UKPDS) Brain Bank criteria (16). All of the patients also met the CAPSIT-PD criteria (17) permissive to the qualification to bilateral STN-DBS. The patients were divided into three groups: BMT-group (Best Medical Therapy) consisted of 20 patients (56.7 mean age, 11 females, 9 males) treated only with pharmacotherapy through the whole time of observation, DBS-group (Deep Brain Stimulation) consisted of 20 patients (51.1 mean age, 8 females, 12 males) which underwent surgical procedure and pharmacotherapy, POP-group (Postoperative) consisted of 14 patients (51.4 mean age, 7 females, 8 males) which were operated in 30-months median time before the study began (this group was created to estimate a long-term effect of DBS on balance disorders). Demographic data of patients are described in Table 1. All of the patients signed an informed consent. The Ethics Committee of Medical University of Warsaw approved the study. The experiments were conducted in accordance with the ethical standards of the Declaration of Helsinki.

**Table 1:** Study population.

	**BMT-group**	**DBS-group**	**POP-group**
Gender	11 F, 9 M	8 F, 12 M	7 F, 8 M
Age at study beginning	56.7 ± 15.4 years	51.1 ± 15.3 years	51.4 ± 8.7 years
Time of onset	46.3 ± 15.1 years	39.7 ± 13.3 years	40.9 ± 8.3 years
Symptoms' duration time	10.4 ± 4.9 years	11.3 ± 3.9 years	10.5 ± 3.5 years
Time of dyskinesia	1.8 ± 2.6 hours daily	4.9 ± 2.9 hours daily	5.9 ± 2.6 hours daily
OFF time	2.7 ± 1.3 hours daily	4.6 ± 3.2 hours daily	4.4 ± 1.8 hours daily
LEDD—Visit 1	1254.0 ± 511.6 mg	1379.5 ± 510.0 mg	(pre-operative): 1273.2 ± 464.3 mg (post-operative): 585.4 ± 409.7 mg
LEDD—Visit 2	1564.0 ± 542.2 mg	350.3 ± 262.2 mg	555.7 ± 499.6 mg
LEDD—Visit 3	1558.3 ± 622.1 mg	394.5 ± 319.2 mg	762.9 ± 589.8 mg
UPDRS III OFF—Visit 1	32.3 pts	34.1 pts	39.5 pts
UPDRS III ON—Visit 1	12.8 pts	11.5 pts	10.2 pts
UPDRS III OFF—Visit 2	37.0^*^ pts	42.9^*^ pts	43.0 pts
UPDRS III ON—Visit 2	12.8 pts	7.9 pts	10.8 pts
UPDRS III OFF—Visit 3	41.7^*^ pts	45.1 pts	47.2 pts
UPDRS III ON—Visit 3	12.8 pts	9.3 pts	11.4 pts

* *p < 0.05*.

All study patients were examined during 3 visits (V1, V2, V3) in 9 ± 2-months periods. In POP-group, pre-operative demographic data and UPDRS III score were also included to this study in order to enable the comparison between three groups.

The balance tests and posturographic assessment were performed by physiotherapist experienced in movement disorders. The parametric evaluation included:

1. posturographic assessment (stage 1: open eyes, stage 2: closed eyes) and biofeedback analysis on TecnoBody Prokin-M-line stabilometric platform with Prokin 3 software
2. clinical balance tests:
  - quantitative tests: Timed Up and Go (TUG), Dual Task- Timed Up And Go Test (DT-TUG)
  - qualitative tests: Tandem Walking Test (TWT), 180° Tandem Pivot Test (TPT)

The motor assessment of study patients (UPDRS scale and parametric stability evaluation) was performed two times during each visit in BMT-group and preoperative assessment in DBS-group (BMT-ON and BMT-OFF phase) and four times (Total-ON, DBS-ON/BMT-OFF, DBS-OFF/BMT-ON, Total-OFF) during postoperative evaluations (V2, V3) in DBS group and during all visits (V1, V2, V3) in POP group. The neurological examination and UPDRS scale evaluation were performed by neurologist experienced in movement disorders. They were performed after 12-h time of levodopa stopping or 24-h time of stopping of other antiparkinsonian drugs in BMT group and in preoperative assessment in DBS group. The neurological evaluation and UDPRS scale during post-operative assessment were performed after 30-min time of switching off both the stimulators (left and right) with 12-h time of levodopa stopping and with 24-h time of other antiparkinsonian treatment stopping (Table 1).

All patients qualified to surgical treatment, underwent bilateral subthalamic nucleus deep brain stimulation (STN-DBS). Pre-operatively fusion of 1,5T MRI and stereotactic contrast-CT was performed with the use of Stereotactic Planning Software (Brainlab). Then, microrecording (MER) and macrostimulation were conducted using Leadpoint®(Medtronic) followed by macrostimulation evaluated by neurophysiologist and movement disorders neurologist. If motor adverse effects appeared below 2V from the M path and visual sensations appeared below 2V from both paths at +2 bilaterally, microelectrodes were replaced by permanent electrodes (3389-28, Medtronic, Minneapolis, MN) bilaterally. Lateral control X-ray was performed to confirm the location of the electrode to be identical to the microelectrode, then the electrode was locked (Stimlock, Medtronic) at the burr-holes and the scalp wounds were closed. After removal of stereotactic frame, the connection of internal pulse generators (Activa SC, Medtronic, Minnneapolis, MN) to the electrodes was performer under general anesthesia. After 4-weeks time, the stimulators were switched on and tunned in order to start the stimulation without adverse effects. If the stimulation effect was balanced and stable, pharmacotherapy was then slowly reduced (Table 1). There were no observed surgical complications after DBS implantation through the whole time of the study.

Data analysis and statistical assessment consisted of the linear mixed model analysis, which was implemented by the use of LME4 (version 1.1) with intercepts for subjects included as random effects. Pairwise interactions between each fixed factor were included in the model. Tukey contrasts (from lsmeans package, version 2.25) were used to compare results between timepoints and treatments (18). All calculations were performed in statistical computing software R (version 3.3) (19). *P* values < 0.05 were considered significant.

## Results

**Figure 1:**
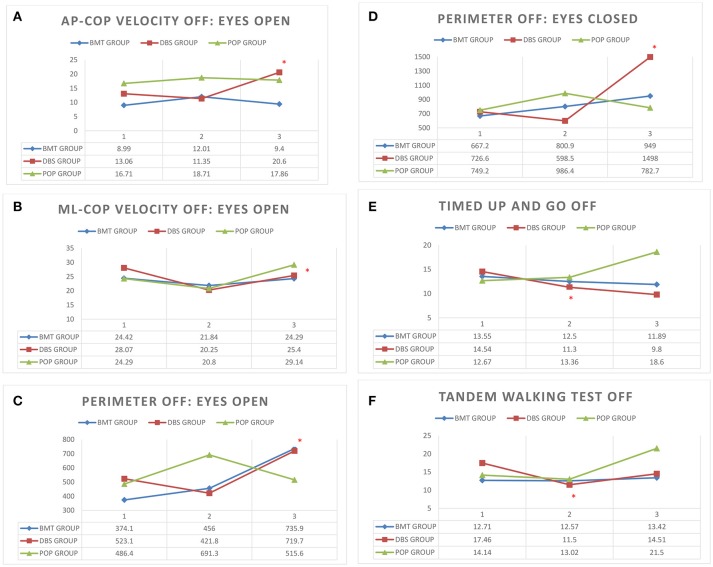
**(A–F)** Posturography and clinical balance tests' parameters in study groups (BMT, DBS, POP) in visits 1–3 (V1, V2, V3) *p < 0.05. **(A)** AP-CoP velocity (mm) with open eyes in Total-OFF phase in study groups (BMT, DBS, POP) in visits 1–3 (V1, V2, V3). **(B)** ML-CoP velocity (mm) with open eyes in Total-OFF phase in study groups (BMT, DBS, POP) in visits 1–3 (V1, V2, V3). **(C)** Perimeter (mm) with open eyes in Total-OFF phase in study groups (BMT, DBS, POP) in visits 1–3 (V1, V2, V3). **(D)** Perimeter (mm) with closed eyes in Total-OFF phase in study groups (BMT, DBS, POP) in visits 1–3 (V1, V2, V3). **(E)** Timed Up And Go test in Total-OFF phase in study groups (BMT, DBS, POP) in visits 1–3 (V1, V2, V3). **(F)** Tandem Walking Test in Total-OFF phase in study groups (BMT, DBS, POP) in visits 1–3 (V1, V2, V3).

Static balance evaluation on posturographic platform revealed alterations in early-post-operative assessment. Static instability in first post-operative Total-OFF phase evaluation (ΔV2-V1 assessment) of DBS group was relatively decreased (*p* > 0.05) in comparison to ΔV3-V2 assessment that revealed statistically significant deterioration of static stability. The mixed model analysis in DBS group showed a slower deterioration (*p* > 0.05) with following significant escalation (*p* < 0.05) in average AP-CoP velocity (average velocity of the center of foot pressure displacement in the anteroposterior direction), average ML-CoP velocity (average velocity of the center of foot pressure displacement in the mediolateral direction), perimeter (length of the path of the center of foot pressure) and ellipse area (area of the greatest sway of the center of foot pressure) in the tests with the eyes open as well as in the tests with eyes closed. The same alterations were not present either in BMT nor in POP-group (*p* > 0.05) (Figure 1).

Clinical balance tests' analysis also revealed different effects in DBS group in Total-OFF phase, which were similar to these observed on posturographic platform: ΔV2-V1 assessment showed improvement in Timed Up And Go tests and Tandem Walking Test (*p* < 0.05) with following deterioration in ΔV3-V2 evaluation in all clinical balance tests (Timed Up and Go tests, Tandem Walking Test, 180° Tandem Pivot Test) (*p* < 0.05). These alterations were not detected in BMT—and POP-group (*p* > 0.05) (Figure 1, Table 2).

**Table 2:** Posturography and clinical balance tests' parameters in study groups: Δ = inter-visit differences.

	**BMT group**				**DBS group**				**POP group**			
**Δ Visit:**	Δ **(V2–V1)**		Δ **(V3–V2)**		Δ **(V2–V1)**		Δ **(V3–V2)**		Δ **(V2–V1)**		Δ **(V3–V2)**	
Phase: Parameter:	ON	OFF	ON	OFF	ON	OFF	ON	OFF	ON	OFF	ON	OFF
Δ AP-CoP velocity [mm/s] /eyes open/	4.07	1.60	2.41	7.71	−17.62	−2.08	0.75	6.75	3.23	5.0	−3.21	−4.71
Δ ML-CoP velocity [mm/s] /eyes open/	1.24	2.91	5.01	6.72	−8.91	−3.31	0.55	9.5^*^	5.53	5.64	−4.18	−5.57
Δ perimeter [mm] /eyes open/	101.3	81.84	132.9	279.9	−505.4	−101.3	26.75	297.9^*^	162.0	204.9	−135.5	−175.6
Δ ellipse area [mm^2^] /eyes open/	360.6	416.7	832.8	773.0	−47.84	−277.9	167.3	214.1	31.15	629.4	−172.5	−467.6
Δ AP-CoP velocity [mm/s] /eyes closed/	5.49	3.09	0.06	5.13	−17.76	−3.49	−2.35	16.4^*^	1.91	7.14	−3.40	−5.5
Δ ML-CoP velocity [mm/s] /eyes closed/	2.75	3.65	3.75	2.87	−9.04	−3.65	−0.4	30.85^*^	5.55	5.21	−2.82	−5.0
Δ perimeter [mm] /eyes closed/	158.0	133.7	72.9	148.2	−502.1	−128.1	−53.1	899.2^*^	129.3	237.1	−115.1	−203.6
Δ ellipse area [mm^2^]/eyes closed/	870.7	1058	400.2	−299.5	583.5	−515.5	89.45	1677^*^	682.0	631.1	−1269	−1169
Δ Timed Up And Go tests [sek]	−1.26	−1.05	0.21	−0.61	0.57	−3.15^*^	0.54	−1.58^*^	0.0	0.69	0.35	5.25
Δ Dual Task—Timed Up And Go test [sek]	−0.91	−1.83	0.23	−0.35	0.85	−8.23^*^	0.20	−0.75	−0.56	−1.49	1.30	5.98
Δ Tandem Walking Test	−2.98	−0.14	1.17	0.85	0.18	−5.93^*^	−0.35	2.97	−0.83	−1.12	4.98	8.52^*^

* *p < 0.05*.

Static and dynamic balance evaluations on posturographic platform and with the use of clinical balance tests, were also performed in Total-ON phase as well as in DBS-ON/BMT-OFF and DBS-OFF/BMT-ON in postoperative assessment in order to estimate the effect of STN-DBS on stability in PD patients. The mixed model analysis of both platform and clinical tests revealed the significant improvement in static and dynamic stability in DBS-ON phases only in V3 evaluation in DBS group (*p* < 0.05), except 180° Tandem Pivot Test assessment which was significantly improved in V1, V2, and V3 Total-ON vs. Total-OFF examination (*p* < 0.05) (Figure 1, Table 2). There was no significant effect of pharmacological treatment on static and dynamic stability within all study groups (*p* > 0.05), except 180° Tandem Pivot Test preoperative (V1) evaluation in DBS group (*p* < 0.05) (Figure 1, Table 2).

## Discussion

Balance disorders are one of the most debilitating motor deficits in PD patients, which increase during the disease progression (20). STN-DBS has been initially shown to have a modest effect on static and dynamic stability (5–10) but long-term studies revealed more conflicting results (11–15, 21). Our study, for the first time, evaluated the long-term effect of STN-DBS on gait and postural instability in Total-OFF phase in PD patients what allows to estimate the possible modulatory effect of STN-DBS on stability disorders progression in PD in comparison to only-pharmacologically treated patients. We revealed the possible modulatory effect of STN-DBS on static and dynamic balance disorders in first post-operative 9-months period with following deterioration in consecutive months. This phenomenon has not been described yet, as our analysis was performed in Total-OFF phase unlikely to previous long-term studies (12, 14, 15), which mainly used UPDRS III examination in postural evaluation rather than posturographic platform or clinical balance tests.

The impact of STN-DBS on static and dynamic balance disorders in PD patients is not clearly established. One of hypotheses is, that the post-operative effect of STN-DBS on balance amelioration may be secondary due to decrease of dyskinesia and motor fluctuations (22). The other authors postulate that STN-DBS can (at least partially) restore functionally the dopaminergic systems (23, 24) and the instability amelioration is than the secondary effect to the decrease of increased STN neuronal activity, burst type activity and abnormal oscillations (25, 26) or due to changes within the entire cortico-striato-pallido-thalamo-cortical system (26, 27). The other hypothesis based on animal models claims that STN stimulation may induce locomotion per direct electrical stimulation of corticobasal locomotor control structures (28). More recent studies showed the possible functional connectivity between STN and sensorimotor and frontoparietal cortical regions' disruption in PD patients with freezing of gait (29) which might be improved directly by STN-DBS electrical effect on the cortico-striato-pallido-thalamo-cortical system and explain the declining impact of STN-DBS on balance disorders in long-term observations (11–14), also observed in our study. Some studies also describe the frequency-dependent effect of STN-DBS on balance instability, with noticeable improvement in low-frequency stimulation (30–32). We have not allocated DBS patients to low- and high-frequency stimulation subgroups as the purpose of this study is to establish the impact of the STN-DBS surgery and long-term electrical stimulation in Total-OFF treatment phase, not to estimate the effect of STN-DBS ON-stimulation on the motor improvement of PD patients, what has been previously reported (30–32).

To conclude, our study revealed, for the first time, the modulatory short-term gait and postural instability improvement with following deterioration of balance disorders in PD patients after STN-DBS surgery. The long-term effect of STN-DBS has not been detected, similarly to lack of noticeable effect of levodopa and other dopaminergic treatment.

## Ethics statement

This study was carried out in accordance with the recommendations of Bioethics Committee of Warsaw Medical University with written informed consent from all subjects. All subjects gave written informed consent in accordance with the Declaration of Helsinki. The protocol was approved by the Bioethics Committee of Warsaw Medical University.

## Author contributions

SS, AF, and DK contributed conception and design of the study. SS, MK, IP, KG, TM, AP, JD, MF, and PH organized the database. SS and DK performed the statistical analysis. SS, AF, AP, and DK wrote the first draft of the manuscript. All authors contributed to manuscript revision, read, and approved the submitted version.

### Conflict of interest statement

The authors declare that the research was conducted in the absence of any commercial or financial relationships that could be construed as a potential conflict of interest.
